# Endosymbiosis morphological reorganization during metamorphosis diverges in weevils

**DOI:** 10.1080/19420889.2020.1840707

**Published:** 2020-11-02

**Authors:** Justin Maire, Bessem Chouaia, Anna Zaidman-Rémy, Abdelaziz Heddi

**Affiliations:** a Univ Lyon, INSA-Lyon, INRA, BF2i, UMR0203, Villeurbanne, France; b Current address: School of Biosciences, The University of Melbourne, Melbourne, VIC, Australia; c Dipartimento di Scienze Agrarie e Ambientali (Disaa), Università Degli Studi di Milano, Milan, Italy; d Current address: Dipartimento di Scienze Molecolari e Nanosistemi (DSMN), Università ca’ Foscari di Venezia, Venice, Italy

**Keywords:** Symbiosis, bacteriome, metamorphosis, weevil, dryophthoridae, evolution, insect, rhynchophorus, nardonella, sitophilus

## Abstract

Virtually all animals associate with beneficial symbiotic bacteria. Whether and how these associations are modulated across a host’s lifecycle is an important question in disentangling animal-bacteria interactions. We recently reported a case of complete morphological reorganization of symbiosis during metamorphosis of the cereal weevil, *Sitophilus oryzae*. In this model, the bacteriome, a specialized organ that houses the intracellular bacterium *Sodalis pierantonius*, undergoes a two-phase remodeling program synchronously driven by host and endosymbiont, resulting in a localization shift and the formation of multiple new bacteriomes. Here, we provide comparative data in a closely-related coleopteran, the red palm weevil *Rhynchophorus ferrugineus*, which is associated with the ancestral endosymbiont *Nardonella*. Using cell imaging experiments, we show that the red pal weevil bacteriome remains unchanged during metamorphosis, hence contrasting with what we reported in the cereal weevil *S. oryzae*. These findings highlight the complexity and divergence of host-symbiont interactions and their intertwining with host development, even in closely-related species.

**Abbreviations**: DAPI: 4′,6-diamidino-2-phenylindole; FISH: Fluorescence *in situ* hybridization; T3SS: Type III secretion system.

## Main text

Symbiotic associations between animals and bacteria are present across all branches of the tree of life, in all ecological niches, and represent a major evolutionary driving force [1–3]. Symbiosis is particularly prevalent in insects, which often associate with intracellular bacteria (endosymbionts) that complement their diet and allow them to thrive on unbalanced niches, such as plant sap or cereals [4,5]. How endosymbiosis and host development are connected is a question of great interest to assess the full impact of bacterial partners on host biology [6]. In insects, a particularly important developmental period is the pupal stage: following larval development, holometabolous insects go through a complete internal and external morphological reorganization, metamorphosis, before emerging as adults [7,8]. Hence, endosymbiosis needs to be both maintained through this drastic morphological changes, and adapted to the physiological needs of the adult [9].

We recently addressed this question using the mutualistic association between the rice weevil *Sitophilus oryzae* and the bacterium *Sodalis pierantonius* [10–12]. *S. pierantonius* is housed in specialized host cells, bacteriocytes, that group together in an organ, the bacteriome [4,12,13], and provides cereal weevils with vitamins and amino acids that are scarcely present in cereals [11,14,15]. Through fluorescent imaging techniques, we showed that, as the insect’s internal organization changes during metamorphosis, the bacteriome is also completely remodeled [16]. The larval bacteriome, a unique organ located around the foregut, dissociates at the onset of metamorphosis, and bacteriocytes migrate along the midgut epithelium [16]. In pupae, bacteriocytes stop their migration around clusters of putative stem cells, positioned at regular intervals along the midgut [16]. At this stage, bacteria were seen entering uninfected stem cells, and electron microscopy further revealed the presence of *S. pierantonius* within the nuclei of these stem cells [16]. This was followed by the formation of multiple new bacteriomes at the apex of mesenteric ceca [16]. Using a dual-RNAseq strategy specifically tailored for the *Sitophilus-Sodalis* association, we showed host transcriptomic modifications in genes associated with cell adhesion and motility, as well as cytoskeleton reorganization [16]. Furthermore, bacterial genes encoding the flagellum apparatus and the type III secretion system (T3SS) were upregulated in pupae [16]. This indicates that *S. pierantonius* transiently adopts an infectious behavior, potentially allowing the bacterium to infect stem cells and, transiently, their nuclei, and to trigger their differentiation into bacteriocytes. Interestingly, it was previously shown that, following metamorphosis, endosymbiont density massively increases during one week, whereupon endosymbionts and bacteriocytes are rapidly eliminated and recycled [14]. Endosymbiont growth was shown to match increasing needs for aromatic amino acids used by *S. oryzae* to rapidly build a strong cuticle following final ecdysis [14]. Hence, we hypothesized that the bacteriome reorganization during metamorphosis is a morphological optimization for the highly demanding and dynamic adult stage, allowing for the growth of the symbiotic population and more efficient host-symbiont metabolic exchanges. This morphological remodeling, synchronously driven by host and endosymbiont transcriptomic changes, shows the extent to which endosymbiosis is integrated with both host developmental processes and metabolic needs.

**Figure 1.**
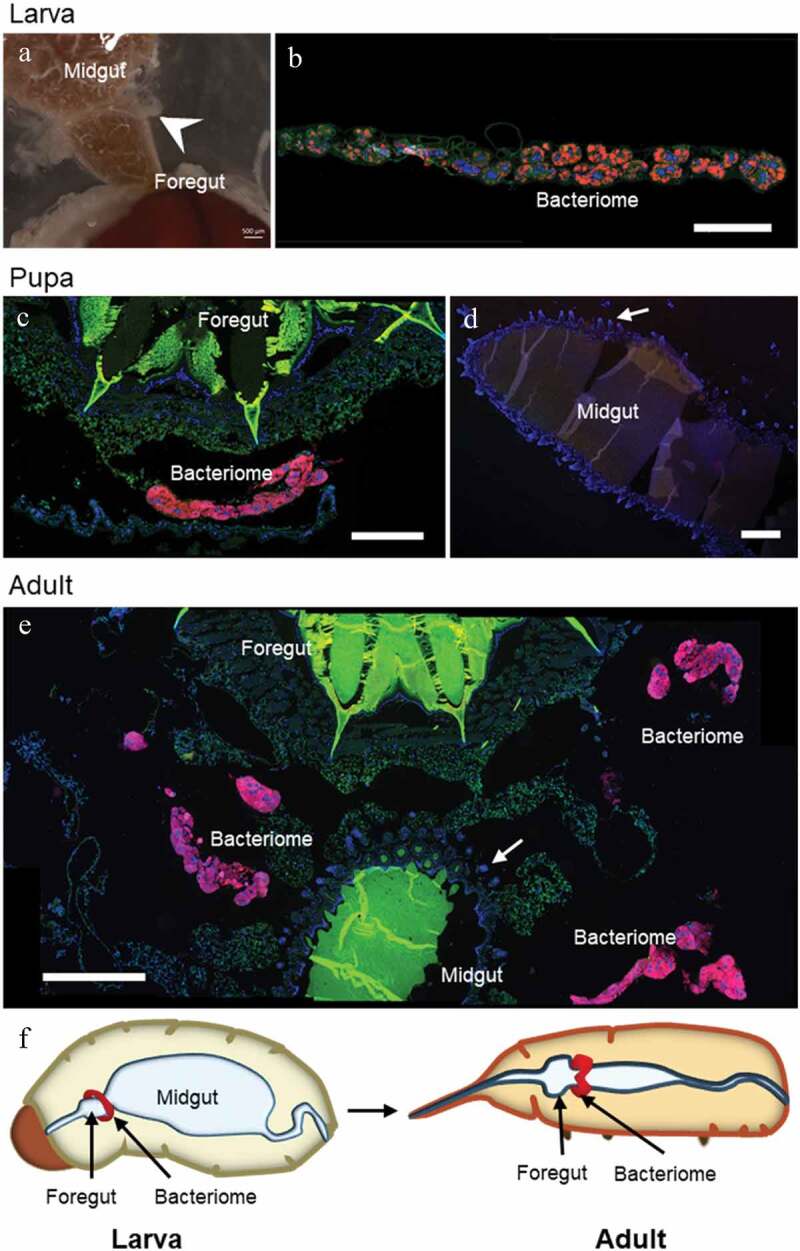
**Bacteriome morphology throughout metamorphosis in *Rhynchophorus ferrugineus***, in the final larval instar (a-b), pupae (c-d), and young adults (e). **A**: Image of a dissected larval gut, with the collar-shaped bacteriome (white arrowhead) attached to the foregut-midgut junction. It can be seen detached and uncoiled in (b). **B-E**: Photos from FISH experiments targeting *R. ferrugineus’* endosymbiont, *Nardonella*. The bacteriome does not change in morphology or localization along metamorphosis: it remains a collar-shaped organ at the foregut-midgut junction. Mesenteric ceca can be seen forming in the midgut (white arrows in D, E), but no bacteriocyte is observed in these areas, unlike in *S. oryzae*. Red: *Nardonella;* green: autofluorescence; blue: DAPI. Scale bar: 250 µm for B, C; 500 µm for A, D, E. **F**: Schematic representation of the bacteriome in a larva and an adult

**Figure 2.**
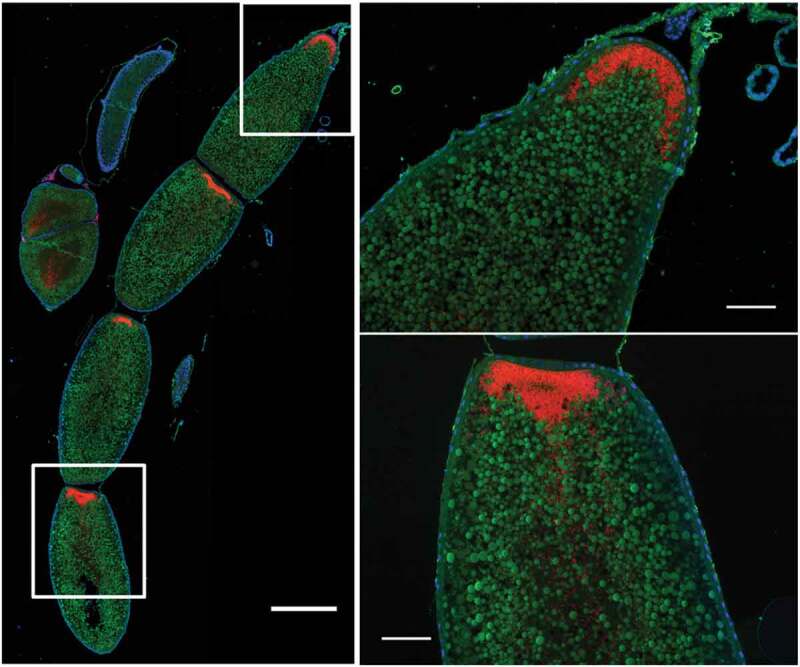
***Nardonella*-infected oocytes in *Rhynchophorus ferrugineus***. Red: *Nardonella*; Green: Autofluorescence; Blue: DAPI. The left image represents four oocytes in a female ovariole, observed in fluorescence microscopy following FISH treatment against the endosymbiont *Nardonella*. Scale bar is 0.5 mm in the left photo and 100 µm in the close-ups

Whether bacteriome reorganization is conserved across species remains elusive. Data from two other *Sitophilus* species, *S. zeamais* and *S. granarius*, show that the process described in *S. oryzae* is conserved within these cereal-feeding species [14]. However, most histological data published so far on related insects are limited to larvae [17,18], and did not address the fate of symbiotic organs during metamorphosis. Here, we provide additional imaging data from a closely-related coleopteran of the Dryophthoridae family, the red palm weevil *Rhynchophorus ferrugineus*. The red palm weevil is one of the most damaging palm-trunk borring pest worldwide, and feeds mainly on sap and tender tissues [19]. *R. ferrugineus* harbors the endosymbiont *Nardonella*, the ancestral endosymbiont of the Dryophthoridae family to which *Sitophilus* weevils belong [17,20,21]. Wild *R. ferrugineus* were collected and fluorescence *in situ* hybridization (FISH) with a *Nardonella-*specific probe was conducted on dissected bacteriomes and guts. Surprisingly, while the gut underwent morphological reorganization during *R. ferrugineus’* metamorphosis, the bacteriome remained unchanged and unmoved, collar-shaped at the foregut-midgut junction, from larva to imago (Figure 1). This markedly contrasts with our previous observations in *Sitophilus*. Nonetheless, endosymbionts were observed within the oocytes (Figure 2), similarly to *Sitophilus* [22], indicating that transmission and, potentially, bacteriome formation during embryogenesis, are conserved.

Both *Nardonella* and *S. pierantonius* are known to participate in host cuticle formation by providing aromatic amino acids [14,21], a feature that is shared across other beetle-bacteria associations [23,24]. In coleopterans, a thick and strong cuticle is essential to protect adults against predators, desiccation, and other environmental conditions [23]. However, the ‘urgency’ in cuticle formation that was shown in *S. oryzae* (*i.e*. a rapid increase in endosymbiont density associated with a rapid cuticle synthesis) has not been proven in *R. ferrugineus*. Previous work on the related weevil *Pachyrhynchus infernalis*, also harboring *Nardonella*, showed signs of cuticle synthesis 35 days after final ecdysis, as evidenced by changes in cuticle color and thickness [21], a much longer timespan than that observed in *S. oryzae* (*i.e*. 7 days, 14). A similar timespan (28 days) was observed for cuticle synthesis in *Pachyrynchus sarcitis kotoensis* [25]. Interestingly, the cereal weevil *S. granarius* also exhibits a slower cuticle synthesis and symbiont dynamics than *S. oryzae* (~15 days until cuticle completion, versus 7 days in *S. oryzae*) [14]. This was correlated with a less contrasted symbiont dynamics in *S. granarius* associated with a ‘flattened’ endosymbiont density curve [14]. Unlike *S. oryzae* and *S. zeamais, S. granarius* is not found in field cereal crops but instead in grain storerooms of temperate countries, and only possesses vestigial wings preventing this insect from flying between different sites [26]. It was hypothesized that this less demanding lifestyle in a relative stable environment does not require a rapid cuticle synthesis [14]. As *Pachyrhynchus* spp. are flightless [27] and *R. ferrugineus* shows limited dispersion in young adults [28], it is likely that these more sedentary lifestyles have resulted in relaxed constraints on the speed of cuticle synthesis following adult metamorphosis, similarly to *S. granarius*. Thus, we speculate that the low endosymbiont dynamics and density, and the reduced host-symbiont metabolic exchanges, might be adapted to the slower cuticle synthesis, and hence that a morphological reorganization of the bacteriome may not be needed in these species.

It is noteworthy that *Nardonella*’s reduced genome lacks the genes encoding the T3SS and the flagellum apparatus [21], and potentially the ability to infect new cells, which might be crucial for the morphogenesis of multiple bacteriomes in *Sitophilus* [16]. Nonetheless, we showed here that *Nardonella* is able to enter oocytes, suggesting the existence of other mechanisms enabling this endosymbiont to enter host cells. Studies in other associations have indicated that various mechanisms have been selected along evolution ensuring endosymbiont transmission to the next generation. This includes exo-/endocytotic processes, which was described in the pea aphid/*Buchnera* association where *Buchnera* also lacks a T3SS [29], maternal bacteriocyte content transfer as observed in the ant *Cardiocondyla obscurior* [30], or the integration of a full, unique bacteriocyte inside the oocytes, as described in whiteflies [31,32]. From our observations, we can likely exlude the latter mechanism in the *R. ferrugineus/Nardonella* association; however further investigation will be required to test the potential implication of the two former mechanisms, or to highlight the selection of another mechanism in this association.

In conclusion, this work highlights the diversity of host-symbiont interactions and morphological features of endosymbiosis in insects of the same family, echoing previous work performed in closely-related stinkbugs [33]. Despite being phylogenetically very close, endosymbiosis in *S. oryzae* and *R. ferrugineus* shows drastically different developmental profiles: both bacteriome morphology and endosymbiont density are highly dynamic in *S. oryzae*, while *R. ferrugineus* exhibits higher stability. While the roots of these dissimilarities are unknown, a lower necessity for *R. ferrugineus* to quickly complete a cuticle is a hypothesis worth investigating. Detailed comparative work on symbiotic and asymbiotic coleopterans should be conducted to fully understand the extent of symbiotic contribution to cuticle synthesis and how it has impacted coleopteran evolution and diversification.

## Material and methods

*R. ferrugineus* weevils were collected in the wild, on Italian palm trees. Both lavae and adults were kept on an apple diet at 28°C until dissection. Insect organs (bacteriomes, guts, ovaries) were dissected in sterile phosphate buffer saline and fixed in 4% paraformaldehyde. Samples were processed and sectioned for histological work as previously described [34].

FISH was performed as previously described [35]. Three individuals were observed per stage (larva, pupa, adult). Probe sequence for *Nardonella* was: TAMRA-CGCGAGAATGAGCAAATCTT (10 µg/mL). Observations, image acquisition and treatment were performed as previously described [34]. Figure 1b-1e and left panel of Figure 2 are mosaics of several photos fused together using the ‘Stitching’ plugin in ImageJ.
